# Complete Genome Sequence of *Sphingomonas* sp. Strain NIC1, an Efficient Nicotine-Degrading Bacterium

**DOI:** 10.1128/genomeA.00666-16

**Published:** 2016-07-14

**Authors:** Xiongyu Zhu, Weiwei Wang, Ping Xu, Hongzhi Tang

**Affiliations:** State Key Laboratory of Microbial Metabolism, and School of Life Sciences & Biotechnology, Shanghai Jiao Tong University, Shanghai, China

## Abstract

*Sphingomonas* sp. strain NIC1, an efficient nicotine-degrading bacterium, was isolated from tobacco leaves. Here, we present the complete genome sequence of strain NIC1, which contains one circular chromosome and two circular plasmids. The genomic information will provide insights into its molecular mechanism for nicotine degradation.

## GENOME ANNOUNCEMENT

*Sphingomonas* sp. strain NIC1, isolated from tobacco leaves, has the ability to degrade nicotine. It can utilize nicotine as a sole carbon and nitrogen source to grow. To date, none of the genomes in *Sphingomonas* with a nicotine-degrading ability have been fully sequenced. To further elucidate the mechanism of nicotine degradation, we present here the complete genome sequence of strain NIC1, which may help to understand the evolution of the nicotine-degrading microorganisms.

The complete genome was sequenced by the PacBio RSII sequencer (Pacific Biosciences, CA). The generated sequencing reads were *de novo* assembled using the Hierarchical Genome Assembly Process (HGAP) protocol RS assembly 2 in SMRT Analysis version 2.3 (Pacific Biosciences [https://github.com/PacificBiosciences/SMRT-Analysis]). The total size of the genome is 3,660,563 bp and is composed of one circular chromosome and two circular plasmids, with G+C contents of 67.4%, 64.5%, and 65.2%, respectively. The coding regions that cover 88.63% of the genome (3,244,506 bp) encode 3,538 proteins. The genome also encodes six rRNAs, 51 tRNAs, and 12 noncoding RNAs (ncRNAs), all of which make up 0.434% of the genome.

Through genome annotation of the *Sphingomonas* sp. strain NIC1 sequence, 22 genes related to the metabolism of aromatic compounds were predicted in the chromosome. The key genes in nicotine degradation, such as *vppB* and *vppD* in *Ochrobactrum* sp. strain SJY1 (1), were annotated in the genome sequence of strain NIC1, consistent with a variant pathway of the pyridine and pyrrole pathway (VPP pathway). More genes associated with nicotine degradation were predicted near these two genes. Genomic islands were identified in the genome of strain NIC1 near the predicted genes of nicotine degradation, which may elucidate the mechanism of the VPP pathway from the aspect of evolution (2). In addition, according to the genomic analysis, some genes relate to the degradation of naphthalene, an aromatic pollutant in the chemical industry. The genes associated with chlorocyclohexane and chlorobenzene degradation were also annotated in the genome of strain NIC1. Moreover, 105 genes related to motility and chemotaxis were found in genome of strain NIC1, which may enhance the motile ability of bacteria to locate and degrade chemical compounds (3).

### Nucleotide sequence accession number.

The genome sequence of *Sphingomonas* sp. NIC1 has been deposited in GenBank under the accession no. AJLO00000000.
